# Repurposing liraglutide to the management of DSS-induced colitis: a potential for promoting autophagy

**DOI:** 10.1007/s00210-025-04339-w

**Published:** 2025-06-05

**Authors:** Ahmed Atef Saadoun, Alia Hamed Abdelsattar, Amro Hatem Elsaid, Eslam Abdelaziz Abdelaleam, Hazem Khaled Abdelkader, Hend Mohamed Ibrahim, Merna Sabri Saad, Moumen Said Ellawi, Rana Elshahawi Elsaid, Aya Maghrabia, Dina Ibrahim, Nehal M. Ramadan

**Affiliations:** 1 Mansoura Manchester Medical Program, Faculty of Medicine, Mansoura University, Mansoura, 35516 Egypt; 2 Program of Medicine, Faculty of Medicine, Mansoura University, Mansoura, 35516 Egypt; 3 Medical Experimental Research Center (MERC), Faculty of Medicine, Mansoura University, Mansoura, 35516 Egypt; 4 Pathology Department, Faculty of Medicine, Mansoura University, Mansoura, 35516 Egypt; 5 Clinical Pharmacology Department, Faculty of Medicine, Mansoura University, Mansoura, 35516 Egypt; 6 Clinical Pharmacology Department, Program of Medicine, Mansoura National University, Mansoura, Egypt; 7 Department of Clinical Pharmacology, Horus University in Egypt (HUE), New Damietta, Egypt

**Keywords:** Ulcerative colitis, Paneth cell metaplasia, Liraglutide, Autophagy

## Abstract

Ulcerative colitis (UC) is a chronic inflammatory bowel disease (IBD) that typically requires long-term drug treatment. The emerging role of glucagon-like peptide 1 (GLP-1) and its receptor agonists in gastrointestinal diseases has drawn much attention. A mouse model of acute colitis was conducted using a 7-day protocol of dextran sulfate sodium (DSS, 3% w/v in drinking water) administration. The efficacy of liraglutide (a GLP-1 receptor agonist) in treating DSS-induced colitis and its accompanying Paneth cell metaplasia was examined and correlated to its impact on the levels of colonic autophagy. Liraglutide effectively ameliorated colitis symptoms in mice. Dose-dependent improvements in disease activity index (DAI), colon length, and histopathological scores of inflammation and mucosal damage were evident in the liraglutide-treated groups compared to the DSS group. In addition, liraglutide reduced the expression of lysozyme and suppressed colonic Paneth cell metaplasia induced by DSS. Notably, liraglutide also enhanced levels of colonic autophagy. Direct visualization of autophagosomes and autolysosomes under an electron microscope along with p62 accumulation following co-treatment with chloroquine supports the role of liraglutide as an autophagy enhancer. Liraglutide ameliorated DSS-induced colitis and prevented Paneth cell metaplasia possibly via enhancing colonic autophagy. Our findings strongly signify liraglutide as a therapeutic option for UC.

**Supplementary Information:**

The online version contains supplementary material available at 10.1007/s00210-025-04339-w.

## Background

**Inflammatory bowel disease (IBD)**, with its two clinical phenotypes, Crohn’s disease (CD) and ulcerative colitis (UC), has become a global ailment with a high prevalence in the West and a rapidly increasing incidence in the East (Mak et al. 2020). The pathogenesis of IBD is multifactorial, and its chronic inflammatory nature is yet not fully understood mechanisms (Hibi et al. 2020; Mak et al. 2020).

Several treatment options are available for UC, including 5-aminosalicylic acid (5-ASA), corticosteroids, thiopurines, and TNF-α monoclonal antibodies. Despite showing efficacy in many patients, several problems stemmed from the long-term use of these drugs, whether it is the inconvenient frequent dosing of 5-ASA, the plethora of side effects accompanying long-term corticosteroid use, the cytotoxicity of thiopurines, or the cost barrier and severe side effects of monoclonal antibodies (Kane 2006; Kornbluth & Sachar 2010). Thus, the need to investigate new, safer, and more convenient drugs presents itself as an imperative.

Autophagy is an evolutionary conserved cellular pathway in which intracellular components are degraded and eventually recycled to serve in (a) eliminating aged and damaged cellular organelles and faulty proteins, (b) removing intracellular pathogens, (c) maintaining intracellular homeostasis, and (d) aiding cell survival, especially during times of cell stress (5). Earlier studies have linked polymorphisms in autophagy-related genes (ATGs) to susceptibility and development of IBD in both experimental animals and human populations (Cadwell et al. 2008; Kim et al. 2019). Inducers and suppressants of autophagy have been tested for their effect on the course of IBD, however with variable, sometimes contradicting, results, with some studies indicating that stimulators of autophagy ameliorate the disease (Shao et al. 2021), while others demonstrating improvement after administration of autophagy inhibitors (Wang et al. 2019). Thus, further research is needed to clarify the interactions between autophagy and IBD development.

**Liraglutide** is a glucagon-like peptide 1 (GLP-1) receptor agonist (Neumiller & Campbell 2009) that gained its FDA approval for type 2 diabetes mellitus in 2010, and in 2014, it gained approval for use in obesity (Alruwaili et al. 2021). Given the notable prevalence of obesity and other metabolic disorders among patients with UC (~ 40%) and their negative impact on disease progression (Elangovan et al. 2023), repurposing liraglutide, to the management of colitis seems particularly interesting.

Previous reports have pointed to the role of GLP-1-based therapies in controlling intestinal inflammation (Villumsen et al. 2021). Diabetic patients on GLP-1 receptor agonists or DPP-4 inhibitors tend to have a reduced likelihood of IBD progression compared to those on other anti-diabetic drugs (Villumsen et al. 2021). In the AdTr model of colitis, liraglutide was able to control disease activity possibly by reducing the expression of inflammatory cytokines and chemokines (Bang-Berthelsen et al. 2016).

**Chloroquine**, originally recognized for its antimalarial properties, exerts considerable influence on the immune system. Via alteration of lysosomal pH, chloroquine can impede the processing and presentation of antigens, thus diminishing the activation of immunological cells (Haładyj et al. 2018). In addition to its immuno-modulatory effects, accumulation of chloroquine within lysosomes interrupts the formation of autophagic autolysosomes and interferes with autophagic flux (Ferreira et al. 2021). Chloroquine was previously shown to modulate T cell subset function and/or differentiation, and rectify the balance between TREG and the pathogenic TH17 cells in the mouse model of DSS-induced colitis. Such positive effects were blunted when chloroquine was used in higher concentrations; this was attributed to the intestinal autophagy-inhibiting properties noticed with the higher concentrations (Park et al. 2019).

To our knowledge, no study available has coupled liraglutide’s potential to improve colitis with its autophagy-modulating properties. Therefore, the current study aimed to investigate the impact of liraglutide therapy on DSS-induced colitis in mice, and to specifically explore the autophagy changes accompanying colitis, and the effects of liraglutide therapy on those changes. “Using the same model, we provided evidence that adding liraglutide to chloroquine therapy can address limitations put forth by chloroquine’s autophagy-inhibiting property and permit it to effectively control the overinduction of various forms of inflammatory and immune responses.” The combination of the two drugs can significantly enhance the effectiveness of chloroquine in the treatment of IBD.

## Materials and methods

### Reagents

Liraglutide (VICTOZA® injectable pen; 18 mg/3 mL) was supplied by Novo Nordisk A/S (Bagsvaerd, Denmark). Chloroquine diphosphate salt was purchased from Sigma-Aldrich Co (#C6628; St. Louis, USA). DSS (MW ca 40,000) was purchased from Alfa Aesar (#J63606.22; Ward Hill, MA). Mouse p62 (sequestosome-1; SQSTM1) ELISA kit was obtained from MyBioSource (#MBS3806181; San Diego, CA, USA). Antibody against lysozyme (#61118SS; Rabbit polyclonal) was purchased from Novus Biologicals, LLC (Easter Ave, USA).

### Animals

Eight-week-old C57BL/6L male mice were obtained from Mansoura Experimental Research Center (MERC; Mansoura University, Egypt) and housed under standard laboratory conditions (22 ± 2 °C, 12-h light: 12-h dark cycles). A regular chow diet and water were available as ad libitum throughout the experimental period.

### DSS-induced ulcerative colitis model

**Fig. 1:**
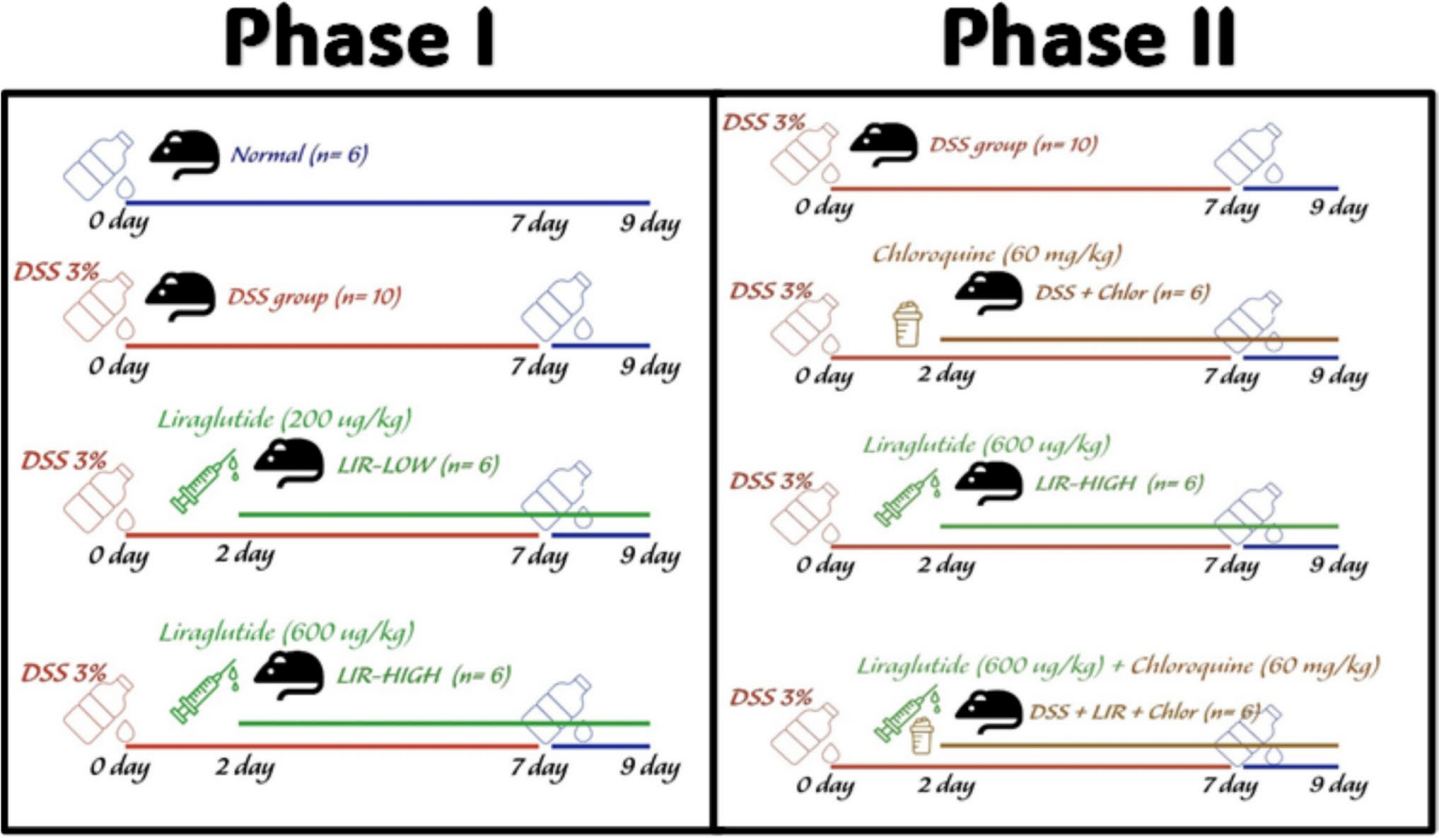
Timeline and experimental design. The DSS groups received a single cycle of drinking water containing 3% DSS (7 days), followed by 2 days of normal drinking water. Other groups underwent a similar induction protocol + administration of liraglutide alone (0.2 and 0.6 mg/kg/day SC) or in combination with chloroquine (60 mg/kg/day PO, phase II) for 7 days, starting 2 days after the onset of DSS induction. DSS, dextran sodium sulfate; LIR, liraglutide; Chlor, chloroquine

Following a single week of acclimation, animals (*n* = 28) were randomly assigned to four groups: normal (received drinking water; *n* = 6), DSS (received 3% DSS in drinking water; *n* = 10), LIR-Low (received 3% DSS + liraglutide 200 µg/kg/day SC; *n* = 6), LIR-High (received 3% DSS + liraglutide 600 µg/kg/day SC; *n* = 6). The acute colitis model was induced by oral administration of 3% DSS (w/v) in the drinking water for 7 days (Dai et al. 2020). Mice were then allowed 2 days of regular water drinking before scarification (Fig. 1). Liraglutide injections were started 48 h after DSS and continued to scarification.

For further assessment of the autophagy modulating effects of liraglutide, a second patch of C57BL/6L mice (*n* = 24) were then assigned to: the DSS group (received 3% DSS in drinking water; *n* = 6), DSS-Chlor (received 3% DSS + chloroquine 60 mg/kg/day SC; *n* = 6), DSS-LIR (received 3% DSS + liraglutide 600 µg/kg/day SC; *n* = 6), DSS-LIR + Chlor (received 3% DSS + liraglutide 600 ug/kg/day SC + chloroquine diphosphate 60 mg/kg/day orally; *n* = 6). Model induction and treatment duration were exactly as mentioned above.

### Assessment of disease activity (severity of colitis)

A Disease Activity Index (DAI) score was calculated daily throughout the experiment to evaluate colitis’s development and treatment’s effect across the different groups. The DAI was a composite of factors, including relative body weight loss (0–4), stool softness (0–4), and the presence of gross blood in the stool (0–4) (Table S1) (Rangan et al. 2019). Two investigators blinded to group labels visually scored stool consistency and bleeding. Acute loss of more than 25% of body weight, along with signs of lethargy and defective grooming, was pre-determined as an indication for humane euthanasia.

After 7 days of liraglutide treatment, mice were weighed and euthanized (pentobarbital sodium, 100 mg/kg IP); spleens and the entire colons between the cecum and rectum were excised. Each colon was thoroughly rinsed with cold normal saline to remove any remaining excreta, and then its length and weight were measured and documented.

### Histopathological analysis

Short segments of the colon (1 cm) were immersed in 10% buffered formalin for 24 h, embedded in paraffin, and sliced into 4-µm sections. The leftover colonic tissues were utilized for electron microscopy or stored immediately at − 80 °C for ELIZA.

Colon sections stained with hematoxylin and eosin (H&E) were observed under a light microscope (Leica, Germany) for signs of mucosal damage (Abdelbaqi et al. 2006). The pathological severity of colitis was blindly scored as the sum of the following: inflammatory infiltrate (0–3), crypt damage (0–3), muscle thickness (0–3), goblet cell depletion (0/1), and presence of crypt abscess (0/1). Hence, the scale for colitis severity ranges from 0 to 11. Three microscopic fields per sample were randomly selected, scored, and averaged.

For immunohistochemistry, tissue sections were deparaffinized, rehydrated, and underwent antigen retrieval in a heated citric acid buffer (10 mM citric acid monohydrate buffer pH 6.0). They were then incubated with the primary antibody against lysozyme enzyme (1/100, overnight at 4 °C). Afterward, they were exposed to an HRP-conjugated anti-rabbit secondary antibody. Ultimately, all sections were stained with DAB stain and then protected with coverslips. High-quality images were obtained using the Touptek USB-2.0 camera (ToupCam SCMOS05000 KPB). A minimum of 15 random fields per group were imaged and quantitatively analyzed using FIJI/IMAGE J v1.53 d software (Media Cybernetics, Inc., Rockville, MD, USA) (Schneider et al. 2012).

### Transmission electron microscopy (TEM)

Colonic tissue specimens (1 × 1 × 1 mm) were cut and rapidly fixed in a cold mixed paraformaldehyde/glutaraldehyde (4 F1G) fixative solution to visualize autophagosomes. The specimens were then dehydrated and embedded in epoxy resin. Semithin Sects. (1-um thickness) were prepared and stained with toluidine blue. Ultrathin Sects. (60–70-nm thickness) were prepared and stained with uranyl acetate and lead citrate. The examination was done using a JEOL JEM-2100 (Japan) TEM at 200 kV (TEM unit located at the Faculty of Agriculture, Mansoura University, Egypt).

### Analysis of p62 levels

Colon tissue samples were homogenized and centrifuged at 15,000 g for 10 min. The levels of p62 in the supernatant were measured using the ELISA technique according to the protocol of the manufacturer.

### Statistical analysis

Data were analyzed using IBM-SPSS software (Version 26), and results were plotted as mean ± standard error (SEM) or as median and range as applicable. After confirming the normality of data distribution, one-way ANOVA followed by Tukey post hoc analysis was used to compare quantitative data among the study groups. The Kruskal–Wallis analysis of variance (ANOVA) followed by Dunn’s-Bonferroni test was used to compare histopathological scores among the separate groups. For any test, results were deemed statistically significant if the *p*-value was less than 0.05.

## Results

### Liraglutide dose-dependently improved colitis symptoms induced by DSS in mice

**Fig. 2:**
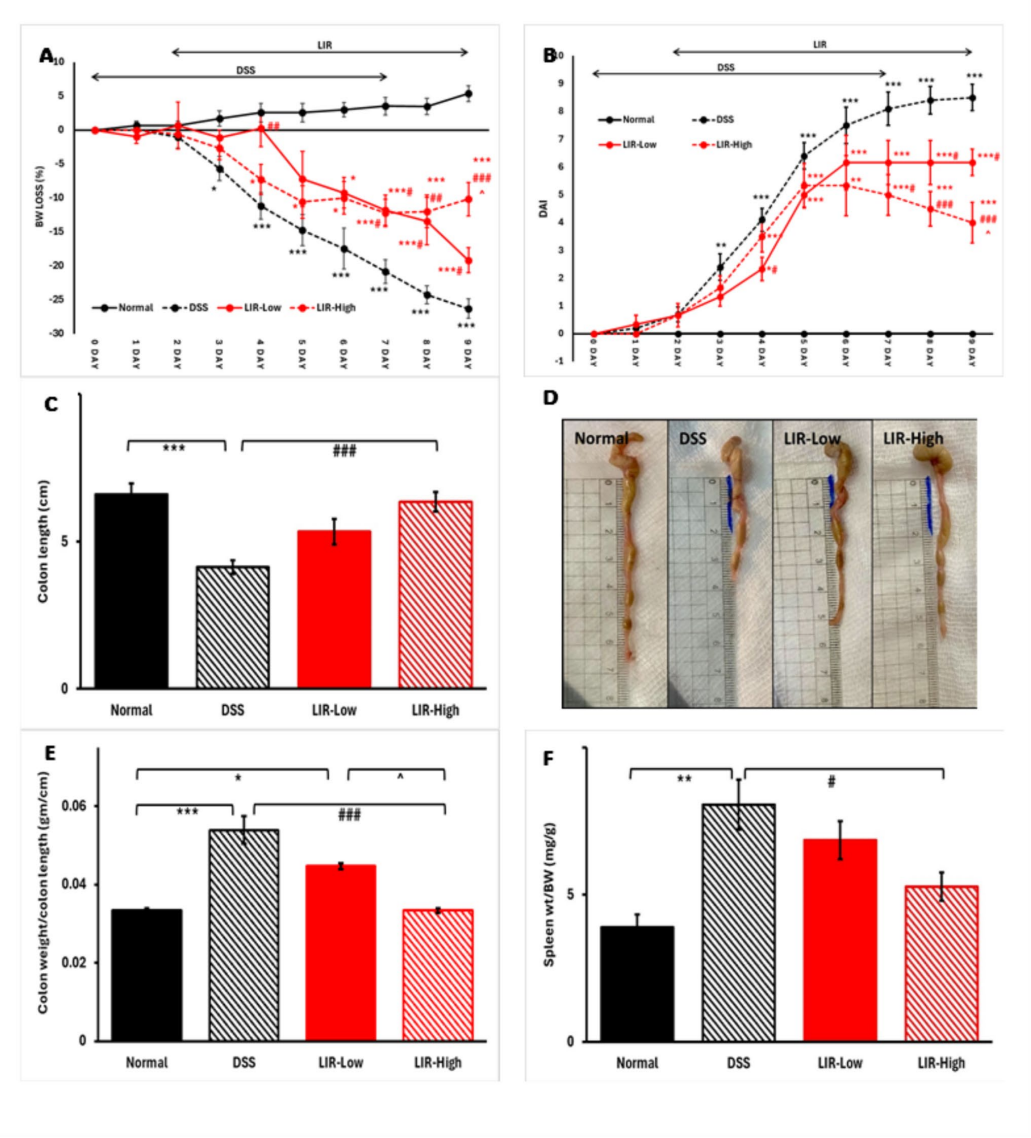
Liraglutide treatment significantly improved clinical signs of DSS-induced colitis (*n* = 6–10). **A** Percentage of body weight reduction and **B** Disease Activity Index (DAI) is presented over the 9-day experimental regimen. **C**, **D** Colon length in CM. **E** Relative colon weight/length in gm/cm. **F** Relative splenic weight/body weight in mg/gm. Data are shown as means ± SEM. Statistical analyses were conducted using one-way ANOVA, followed by Tukey’s post hoc test. *p < 0.05, **p < 0.01, ***p < 0.001, compared to Normal; #*p* < 0.05, ##*p* < 0.01, ###*p* < 0.001 compared to DSS; ^*p* < 0.05 compared to LIR-Low group

Administration of DSS (3% in the drinking water) for 7 days successfully induced colitis in C57BL/6 mice. Clinical indicators of colitis include reduced body weight, loose stools, and blood in stool. Three days following the start of DSS, mice in the DSS group began to show substantial weight reduction, with their body weight remarkably lower compared to the Normal group. Administration of liraglutide in low (0.2 mg/kg) and high (0.6 mg/kg) doses limited the mice’s body weight reduction (Fig. 2A). Of note is that mice started on high-dose liraglutide showed initial weight reduction compared to those started on the lower dose (*p* < 0.005 on the 4 th experimental day); afterward, they began to catch up weight, achieving significantly higher body weights at the end of the experimental period (*p* < 0.05 vs. LIR-Low group).

In harmony, Fig. 2B demonstrates a considerable increase in the DAI scores following DSS administration, which was then suppressed by liraglutide therapy. The initial body weight reduction observed in the LIR-High group was parallel to an initial increase in the DAI compared to the LIR-Low group; the DAI was then decreased in favor of the higher dose group at the end of the experimental period (*p* < 0.05 vs. LIR-Low dose group). This phenomenon was possibly related to the increased incidence of initial dose-dependent gastrointestinal side effects (including diarrhea) with the higher dose of liraglutide.

DSS administration also resulted in a marked reduction in colon length (Fig. 2C and D) and an increase in relative colonic and splenic weights (Fig. 2E and F) compared to normal mice. However, these alterations were significantly improved when liraglutide was administered SC in a high dose (0.6 mg/kg).

### Liraglutide dose-dependently promoted histopathological recovery of DSS-induced colitis in mice

**Fig. 3:**
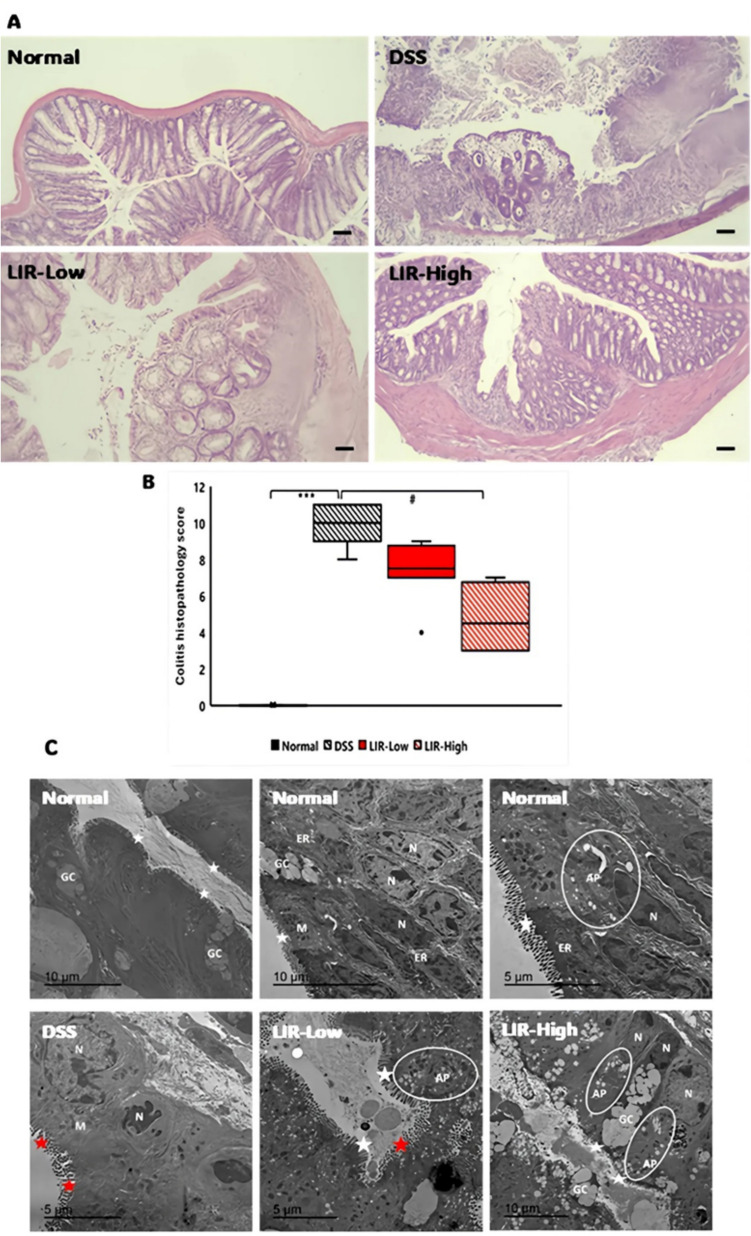
Liraglutide dose-dependently promoted structural/ultrastructural recovery of DSS-induced colitis. **A** In contrast to normal crypt architecture observed in normal mice, colons of the DSS group showed focal ulceration and loss of crypts, marked inflammatory cell infiltrate, and depletion of goblet cells. In a dose-dependent fashion, liraglutide-treated groups exhibited mild to moderate improvement in crypt structure, inflammatory cell infiltrate, muscle thickness, and number of goblet cells. Scale bar = 100 um. **B** Histopathological scores of colons from different experimental groups (*n* = 6). Data are shown as median and range. Statistical analysis was conducted using Kruskal–Wallis analysis of variance (ANOVA) followed by Dunn’s-Bonferroni test. ***p < 0.001, compared to Normal; #*p* < 0.05 compared to DSS. **C** EM examination of normal colonic tissue revealed columnar cells with intact, tightly packed, regularly arranged microvilli (white stars) and oval basal nuclei (N). Goblet cells (GC) with apical mucin granules along with some autophagy vacuole-like structures (AP) were found. In contrast, shrunken columnar cells with degenerated loosely packed microvilli (red stars) were observed in colons from the DSS group. Nuclei (N) appeared electron-dense and indented. Fibroblast-like cells appeared separated with collagen fibers being deposited at the bottom surface of the epithelium. Liraglutide—dose-dependently—improved cellular structure; however, certain areas still exhibit focal degeneration of microvilli in the LIR-Low group (red stars). Small- to medium-sized AP-like structures were abundant in both LIR-Low and LIR-High groups. ER, endoplasmic reticulum

Microscopic examination of colonic samples from the control group (Fig. 3A) revealed a normal histological architecture, with intact crypts and mucosal structures, and no evidence of inflammatory cell infiltration. In contrast, the colonic tissue from the DSS group (Fig. 3A) showed focal ulceration and loss of crypts, along with significant infiltration of inflammatory cells and depletion of goblet cells. Some cells show dysplastic changes with abnormal increases in mitotic activity (Fig. S2A). Administration of liraglutide dose-dependently improved the mucosal structure of the colon, with limited infiltration of inflammatory cells and a decrease in crypt damage (Fig. 3A).

In harmony, histopathological scores of colons from the DSS group showed a substantial increase (Fig. 3B) compared to the Normal group. Nevertheless, therapy with liraglutide effectively averted the increased histopathological score caused by DSS (*p* < 0.05 for the LIR-High group).

### Liraglutide dose-dependently improved colonic ultra-structural aberrations induced by DSS in mice

EM examination of the normal colon showed the typical pattern of columnar cells with oval basal nuclei and tightly packed microvilli (Fig. 3C). However, colons from the DSS group exhibited shrunken columnar cells with electron-dense indented nuclei. Evidence of collagen deposition in the lamina propria was also noted.

Especially true for the LIR-High group, columnar cells displayed a near-normal pattern of regularly arranged microvilli, with rounded or oval nuclei.

### Liraglutide suppressed colonic Paneth cell metaplasia induced by DSS in mice

**Paneth cells** are specialized intestinal epithelial cells known to secrete antimicrobial proteins, such as the lysozyme enzyme (Bel et al. 2017). Innate Paneth cells are primarily found in the crypts of Lieberkühn of the small intestine (5–16 Paneth cells per mouse crypt). They are considered “metaplastic” when observed further down the colon during pathological conditions. Metaplastic Paneth cells are commonly described in IBD, including UC (Simmonds et al. 2014).

**Fig. 4:**
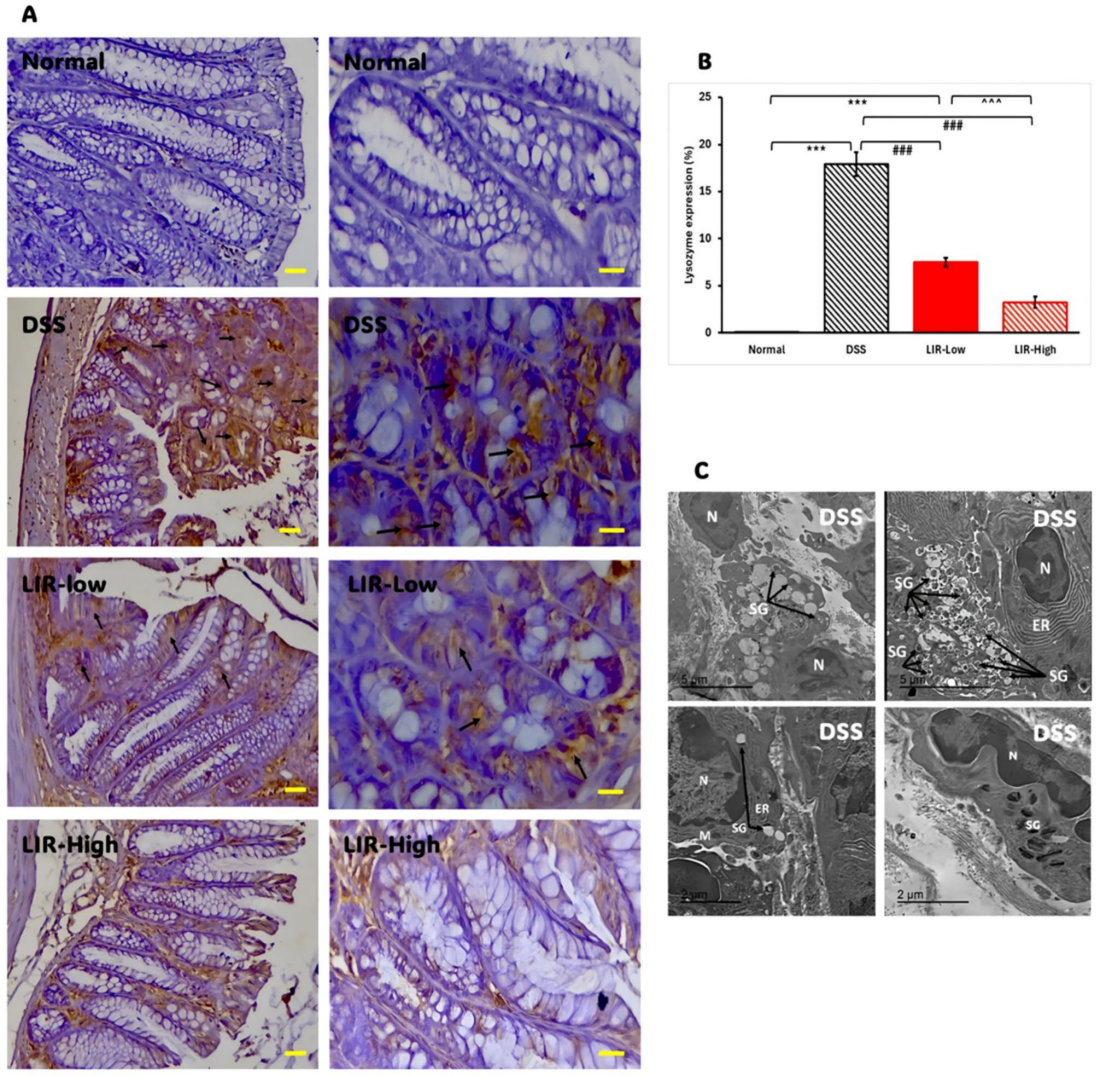
Liraglutide suppressed DSS-induced colonic Paneth cell metaplasia. **A** Immunohistochemical detection of lysozyme in the colon of DSS mice. Note the absence of lysozyme expression in the Normal colon. Active inflammation in the colons of the DSS was associated with enhanced immunoreactive for lysozyme (thin arrows). Not only metaplastic Paneth cells but also many enterocytes are strongly and diffusely immunoreactive for lysozyme. Colons of liraglutide-treated mice showed a dose-dependent decrease in lysozyme staining compared to those on DSS alone. **B** Semiquantitative analysis of lysozyme expression across different experimental groups (*n* = 6–10). Data are shown as means ± SEM. Statistical analyses were conducted using one-way ANOVA, followed by Tukey’s post hoc test. ***p < 0.001, compared to Normal; ###*p* < 0.001 compared to DSS; ^^^*p* < 0.05 compared to LIR-Low group. **C** EM micrographs of metaplastic Paneth cells from the DSS colons. Note the few degenerating mitochondria (M) and aberrant loosely packed secretory granules (SG). ER, endoplasmic reticulum

Immunohistochemical staining of colonic samples from our DSS group confirmed the anticipated increase in the lysozyme signal (Fig. 4A and B), indicating an increase in the number of metaplastic Paneth cells. Nevertheless, the colons of liraglutide-treated mice exhibited a noticeable dose-dependent decrease in lysozyme staining compared to those on DSS alone (Fig. 4B).

Using electron microscopy, metaplastic Paneth cells were best detected in the colons from the DSS group. They showed few degenerating mitochondria, aberrant loosely packed secretory granules, and a significant increase in cytoplasmic vacuolation (Fig. 4C). These findings have previously been reported in Paneth cells of patients with IBD homozygous for the *ATG16L1* autophagy protein susceptibility allele (Dvorak et al. 1980). No Paneth cells could be detected in the normal group’s colons.

### Liraglutide enhanced autophagy in the DSS-induced colitis model

**Fig. 5:**
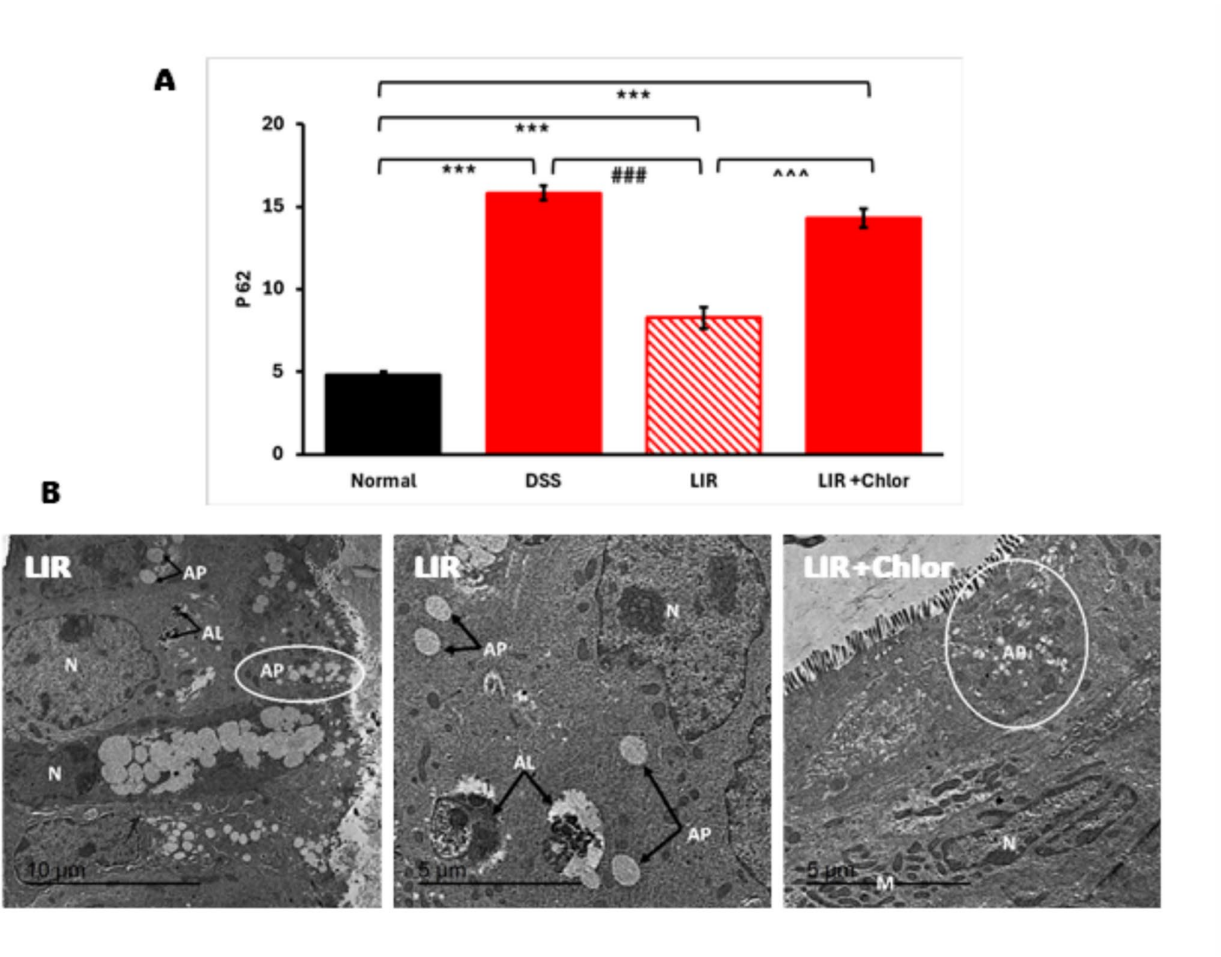
Liraglutide improved DSS-induced colitis by enhancing autophagic flux. **A** Quantitative analysis of the autophagic selective substrate p62 in colons of DSS mice subjected to liraglutide treatment with/without chloroquine (*n* = 6). Data are shown as means ± SEM. Statistical analyses were conducted using one-way ANOVA, followed by Tukey’s post hoc test. ###*p* < 0.001 compared to DSS; ^^^*p* < 0.001 compared to LIR group. **B** EM examination revealed small to medium-sized autophagosome-like structures (AP) in the colons of LIR-Chlor and LIR groups, respectively. Evidence of large autolysosomes (AL) with multiple amorphous inclusion materials was detected in the LIR group only. N, nucleus

Autophagy suppression has been implicated in the development and progression of UC (Kouroumalis et al. 2023). Following the 7-day liraglutide treatment regimen, evidence for an enhanced formation of autophagic vacuoles was detected in the colon of both liraglutide-treated groups (Fig. 3C). To conduct a more thorough examination of this finding, we planned a second set of experiments in which chloroquine (60 mg/kg/day) was used to assess the dynamics of p62/SQSTM1 expression. As shown in Fig. 5A, injection of liraglutide (0.6 mg/kg/day) has resulted in notable reductions in the colonic p62 levels compared to DSS alone. P62, encoded by SQSTM1, acts as a cargo adaptor protein that binds autophagic substrates and targets them to autophagosomes for destruction. During this process, p62 itself undergoes degradation. This explains reductions in the p62 level when autophagy is stimulated (Mizushima et al. 2010). Adding chloroquine to liraglutide allows the pharmacological block of P62 turnover to quantify the autophagic flux amplitude (Klionsky et al. 2016). Substantial accumulation of p62 in the colonic samples of the combination group (LIR + Chlor) further supports the assumption that liraglutide enhances autophagy in the colons of DSS mice (Fig. 5A).

Autophagosome-like vacuoles were observed (Figs. 3C and 5B) and evidence of large autolysosomes with multiple amorphous inclusion materials was detected in the LIR-High group (Fig. 5B).

### Adding chloroquine did not interfere with the liraglutide potential to improve colitis symptoms

Noteworthy, adding chloroquine to liraglutide tended to sustain the improved clinical profile of the LIR (0.6 mg/kg/day) group. Changes in colitis-related body weight loss, DAI, colon length, and relative splenic weight are shown in Fig. S1A–F. In addition, colons from the LIR + Chlor group had comparable histopathological scores compared to the LIR group (Fig. S2A and B).

## Discussion

Autophagy, a finely tuned homeostasis mechanism with yet many blind spots, lays its shadows on various disease processes, including UC. Researchers have linked disturbances in autophagy to the pathogenesis of UC but with contradicting results (Kim et al. 2019; Shao et al. 2021; Wang et al. 2019). The aim of this study, therefore, was to assess the autophagic competency in the colonic tissue of mice treated with DSS to induce a model of UC and to test the effects of liraglutide, a commercially available GLP-1 receptor agonist with potential autophagic enhancement properties.

To confirm the potential benefits of liraglutide in DSS-induced acute colitis, experiments with increasing concentrations of liraglutide (0.2 and 0.6 mg/kg/day) were done. To figure out whether these benefits were related to liraglutide’s ability to enhance autophagy, the high dose of liraglutide (0.6 mg/kg/day) was tested in a replicate set of experiments with or without chloroquine.

A substantial body of evidence implies that impaired cellular autophagy contributes to the development of intestinal inflammation in UC. Aberrations in the expression of autophagy-related genes and their protein translations have been observed in animal models (Kim et al. 2021; Shi et al. 2023) and patient cohorts with UC (Anderson et al. 2011; Kim et al. 2019). Genome-wide association studies (GWAS) have highlighted several autophagy-related genes necessary to enhance susceptibility to bowel inflammatory diseases (de Lange & Barrett 2015; Liu et al. 2015). Examples include, but are not limited to, ATG16L1 (autophagy-related 16 like 1), IRGM (immunity-related GTPase M), ULK1 (unc51–like autophagy activating kinase 1), and LRRK2 (leucine-rich repeat kinase 2) (Foerster et al. 2022)*.* In harmony with such evidence, our study showed that inducing autophagy by liraglutide in a dose-dependent manner contributed to the reduction in symptoms and suppression of inflammation in the DSS-induced UC mouse model.

Concerning its involvement in intestinal immunity, autophagy has been shown to regulate the clearance of invading pathogens by enclosing them inside autophagic vacuoles or promoting their degradation inside autophagosomes. Autophagy also supports intestinal functions of innate and adaptive immunity, including antigen presentation by dendritic cells, cytokine secretion by macrophages, and antimicrobial polypeptide production/secretion by Paneth cells (Kouroumalis et al. 2023).

Paneth cells are typically found in the small intestine and are considered “metaplastic” when observed where they are not generally present, such as in the distal colon (Nakamura et al. 2020). Paneth cell metaplasia is primarily observed in the colonic tissue of IBD patients, including those with UC (Simmonds et al. 2014). Our results highlight a substantial increase in the expression of lysozyme enzyme in the distal colons of DSS mice. This finding was further supported by the presence of metaplastic Paneth cell-like structures (indicated by electron-dense secretory granules) under the EM. A similar phenomenon has been recently reported in the anti-CD40 colitis mouse model (Jayawardena et al. 2024). Colonic Paneth cell metaplasia is believed to be an adaptive protective mechanism where lysozyme and other antimicrobial polypeptides form a barrier against added mucosal infection in an already inflamed bowel (Simmonds et al. 2014). Noteworthy, EM characterization of the metaplastic Paneth cells observed in the colons of our DSS mice revealed few secretory atypical secretory granules with a marked increase in cytoplasmic vesicles; a similar abnormality has been described in the Paneth cells from autophagy-deficient mice (Cadwell et al. 2008; Stappenbeck 2010). The dramatic effect of impaired autophagy on Paneth cell morphology was also reported in CD patients who were homozygous for the ATG16L1 susceptibility allele (Cadwell et al. 2008).

Recently, potential pleiotropic benefits of the anti-diabetic GLP-1 receptor agonist, liraglutide, have been reported in UC, among other inflammatory disorders. In a Danish IBD population, chronic liraglutide therapy reduced the risk of IBD-related adverse clinical events, supporting its favorable influence on disease activity (Villumsen et al. 2021). The sole evidence that liraglutide can directly and promptly modulate the course of UC in humans stems from a case report describing the feasibility of induction of complete remission in a patient with UC via administration of daily subcutaneous liraglutide injections (0.6 mg titrated up to 3.0 mg). The reported side effects were limited to mild nausea associated with dose-up titration (Jeffrey 2019).

Though scarce, experimental evidence does support a promising potential for liraglutide in modulating the intestinal inflammatory milieu characteristic for UC. Via stimulation of the GLP-1Rs, liraglutide could—dose-dependently—increase colonic barrier layer protection and enhance mucosal repair (Bang-Berthelsen et al. 2016). Recently liraglutide’s potential to improve colitis was, in part, attributed to promoting IL 22 production by group 3 innate lymphoid cells (ILC3 s) an effect that was dependent on the colonic microbiome. IL 22 functions to support intestinal epithelial homeostasis, repair, and barrier functions (Sun et al. 2024).

**Fig. 6:**
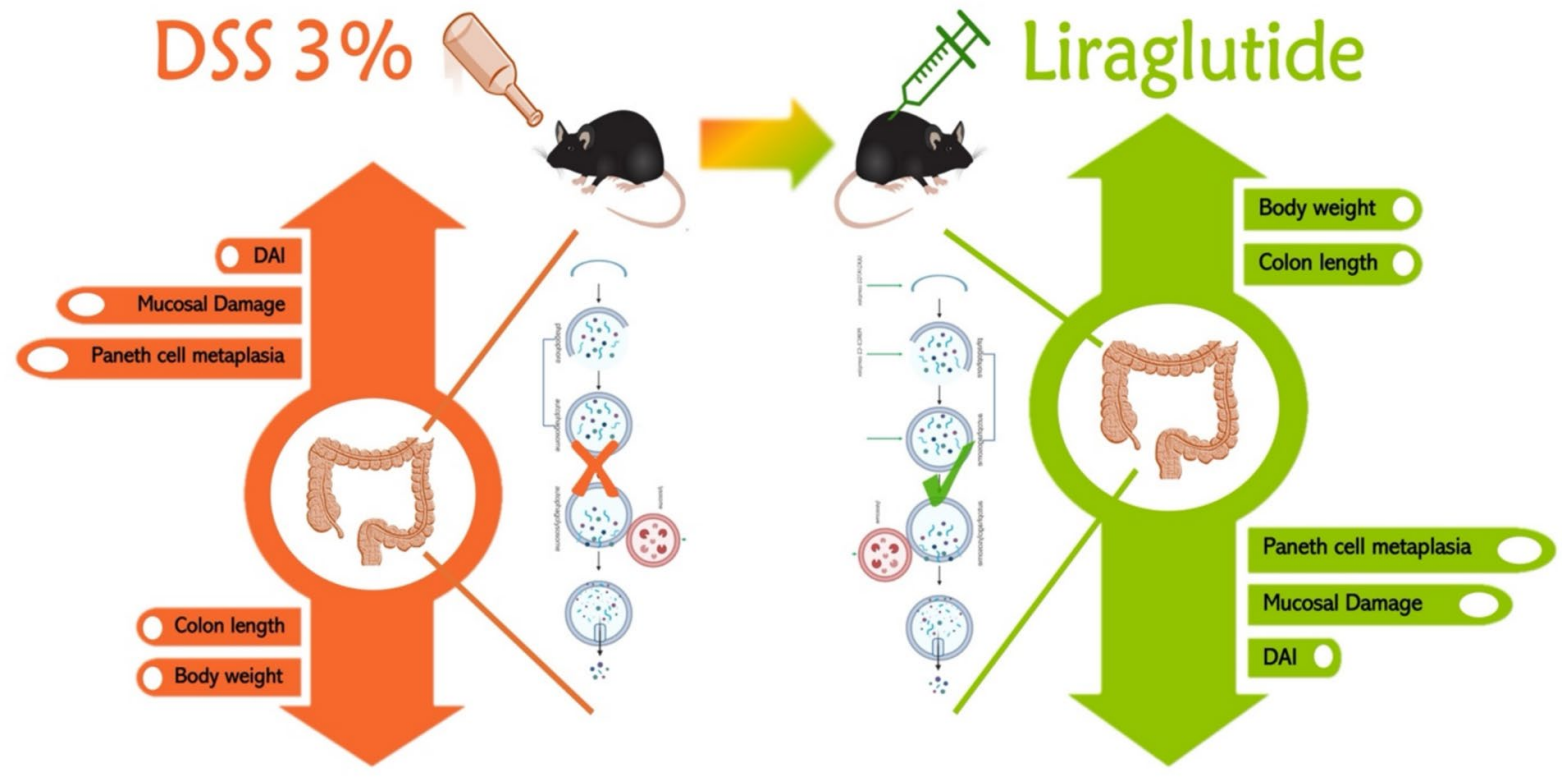
The potential therapeutic role of liraglutide in DSS-induced colitis. Liraglutide improves clinical and histopathological markers of colitis and inhibits Paneth cell metaplasia. Liraglutide enhanced the formation of autophagic vacuoles (autophagosomes and autolysosomes), promoting autophagic competency in the colonic tissue of mice treated with DSS, and facilitating the substantial mitigation of colitis symptoms

Liraglutide has been shown to possess autophagy-enhancing properties in different disease states (Zhang et al. 2021). However, to our knowledge, this is the first study to provide evidence of causation between liraglutide’s potential to enhance autophagy and clinical improvement in the DSS-induced colitis model. We observed significant dose-dependent effects of liraglutide on disease activity, colonic length, and histopathologic inflammation compared with the DSS group. Furthermore, the coincident reduction in P62 expression supports the speculation that liraglutide suppressed the activity of UC by restoring the impaired autophagic flux in the DSS group. Electron microscopic examination also provided evidence of increased autophagosome structures and, more interestingly, increased formation of large cellular degenerative compartments (consistent with lysosomes and autolysosomes) in the high-dose liraglutide-treated group vs. DSS group (Fig. 6).

Earlier data stemming from the work of Zhang et al. (2021) has proven similar autophagy-enhancing potential for liraglutide in the hippocampi of diabetic mice; such potential was also correlated to the liraglutide’s ability to improve the cognitive functions of diabetic mice.

Liraglutide treatment was also associated with a marked dose-dependent reduction in the expression of lysozyme enzyme. Moreover, no evidence of Paneth cell metaplasia could have been detected during the EM examination of the distal colons of liraglutide-treated groups. Overall, these findings support liraglutide’s restorative role in the DSS-induced colitis model.

Being a highly dynamic process, we planned a second set of experiments to accurately assess the autophagic flux in the colonic tissue of liraglutide-treated DSS mice. Assessed indirectly after the addition of chloroquine (an autophagosome-lysosome fusion inhibitor) (Mauthe et al. 2018), enhanced autophagic flux in the colonic tissue of liraglutide-treated mice was confirmed by increased accumulation of P62 in the LIR + Chlor group compared to the LIR group.

It is worth noting that, despite its well-characterized autophagy-inhibiting properties, adding chloroquine to liraglutide (0.6 ug/kg/day) did not impair liraglutide’s efficacy in improving DSS-induced acute colitis in our cohort of mice (Figs. S1 and S2). This can be explained by considering the results of (Nagar et al. 2014). They have suggested that the benefit of chloroquine in DSS-induced colitis stems from blocking the innate immune pro-inflammatory responses. Via inhibiting autophagy in intestinal macrophages (Zhao et al. 2021), chloroquine can block antigen processing by lysosomes, and result in a lack of antigen binding and presentation to class II MHC. However, the same authors have noted that chloroquine’s benefit might be limited by its impact on the intestinal microenvironment, mucosal protection, barrier intactness, and cellular regeneration (Park et al. 2019; Zhao et al. 2021). We believe that such limitation was addressed when liraglutide was added, given liraglutide’s ability to enhance colonic autophagy, increase the barrier layer protection, and selectively repair mucosal tissue.

The current study has the following limitations: First, the specific mechanisms by which liraglutide enhanced intestinal autophagy were not explored. Second, the detailed immunological mechanisms underlying the benefits of adding liraglutide to chloroquine need further emphasis.

## Conclusion

Taken together, we have provided evidence that liraglutide (in a dose-dependent fashion) can protect mice against the development/exacerbation of DSS-induced colitis, at least partially, via inducing colonic autophagy. The addition of liraglutide to chloroquine therapy can address limitations and enhance the effectiveness of chloroquine in the treatment of IBD, though further research is needed on how they beneficially interact together.

## Supplementary Information

Below is the link to the electronic supplementary material.

Supplementary file1 (DOCX 2556 KB)

## Data Availability

All data generated or analyzed during this study are included in this published article [and its supplementary information file]. Raw data are available from the corresponding author upon reasonable request.

## Declarations

### Disclaimer

The funders had no role in the study’s design; in the collection, analyses, or interpretation of data; in the writing of the manuscript; or in the decision to publish the results.
