# Supplementary material for: Repurposing liraglutide to the management of DSS-induced colitis: a potential for promoting autophagy
Source: Naunyn Schmiedebergs Arch Pharmacol. 2025 Jun 5;398(12):17173–85. doi: 10.1007/s00210-025-04339-w (PMC12678552; doi:10.1007/s00210-025-04339-w)
Supplement: Supplementary file 1 — Supplementary file1 (DOCX 2556 KB) [file 210_2025_4339_MOESM1_ESM.docx]

***Supporting information***

**Repurposing Liraglutide to the Management of Colitis: A Potential for promoting autophagy**

Ahmed Atef Saadoun ^1^, Alia Hamed Abdelsattar ^1^, Amro Hatem Elsaid ^1^, Eslam Abdelaziz Abdelaleam ^1^, Hazem Khaled Abdelkader ^1^, Hend Mohamed Ibrahim ^1^, Merna Sabri Saad ^1^, Moumen Said Elawi ^2^, Rana Elshahawi Elsaid ^1^, Aya Maghrabia ^3^, Dina Ibrahim ^4^, Nehal M. Ramadan ^3,5,6,7,^*

^1^ Mansoura Manchester Medical Program, Faculty of Medicine, Mansoura University, 35516, Egypt

^2^ Program of Medicine, Faculty of Medicine, Mansoura University, 35516, Egypt

^3^ Medical Experimental Research Center (MERC), Faculty of Medicine, Mansoura University, 35516, Egypt

^4^ Pathology Department, Faculty of Medicine, Mansoura University, 35516, Egypt

^5^ Clinical Pharmacology Department, Faculty of Medicine, Mansoura University, 35516, Egypt

^6^ Clinical Pharmacology Department, Program of Medicine, Mansoura National University, Egypt

^7^ Department of Clinical Pharmacology, Horus University in Egypt (HUE), New Damietta, Egypt

***** Correspondence: [nehalpharma@mans.edu.eg](mailto:nehalpharma@mans.edu.eg)

**Table of contents**

**Title page** ………… S1

**Table S1** …………. S2

**Figure S1** ………… S3

**Figure S2** ………… S4

**Table S1:** Assessment of disease activity index (DAI)

|  | **Score** | | | | |
| --- | --- | --- | --- | --- | --- |
|  | **0** | **1** | **2** | **3** | **4** |
| **Relative body weight loss** | No weight loss | 1-5% | 6-10% | 11-20% | >20% |
| **Stool softness** | Solid pellet | Soft pellet | Loose stool | Diarrhea | Watery diarrhea |
|  | 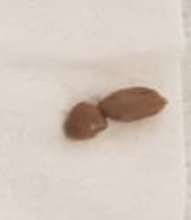 | 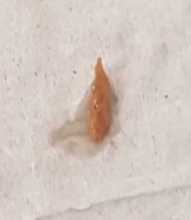 | 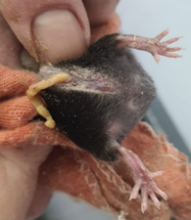 | 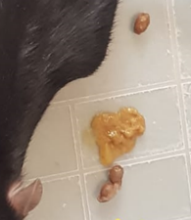 | 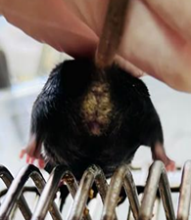 |
| **Presence of gross blood in the stool** | No sign of bleeding | - | Slight bleeding | Bloody diarrhea | Gross bleeding |
|  | 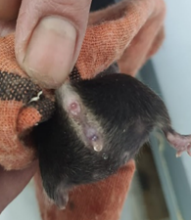 | - | 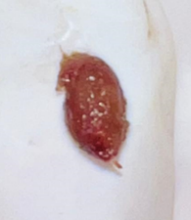 | - | 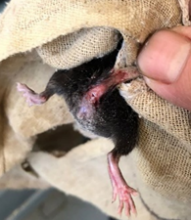 |

**Figure S1**

**
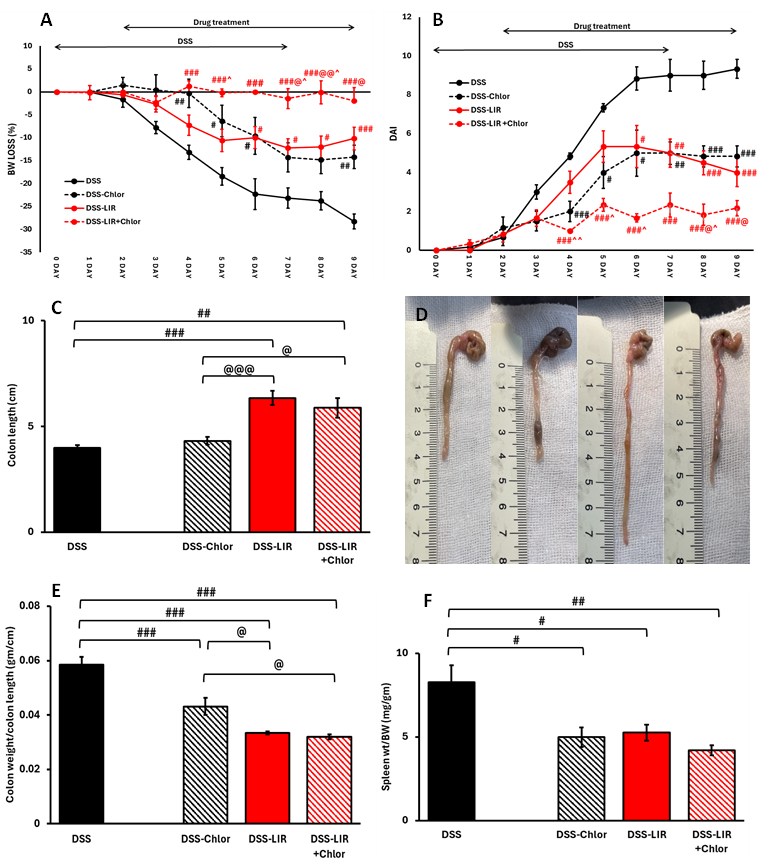
**

**Figure S1: Adding Chloroquine did not antagonize Liraglutide's potential to improve DSS-induced colitis (n = 6).** A: Percentage of body weight reduction and B: Disease activity index (DAI). C: and D: Colon length. E: Relative colon weight/length. F: Relative splenic weight/body weight. #p < 0.05, ##p < 0.01, ###p < 0.001 compared to DSS; @p < 0.05, @@@p < 0.001 compared to DSS-Chlor; ^p < 0.05, ^^p < 0.005 compared to DSS-LIR. Statistical analyses were conducted using *one-way ANOVA*, followed by *Tukey’s* post hoc test.

**Figure S2**


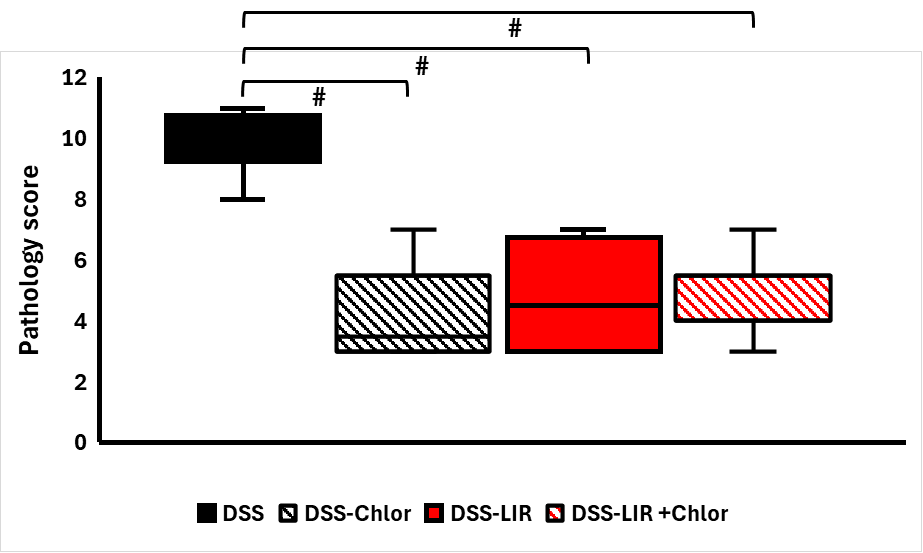

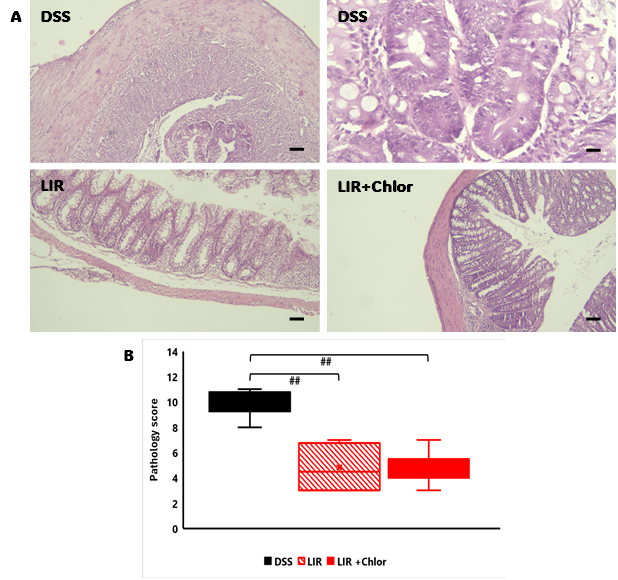


**Figure S2: Adding Chloroquine to Liraglutide did not interfere with histopathological improvements (n = 6).** **A**: In contrast to the DSS group, Liraglutide treatment (0.6 mg/kg/day), either alone or in combination with Chloroquine, has resulted in marked improvement in crypt structure, inflammatory cell infiltrate, muscle thickness, and the number of goblet cells. X:100. **B**: **Histopathological scores of colons from different experimental groups**. Data are shown as median and range. # p<0.05, ## p<0.005 compared to DSS. Statistical analyses were conducted using *Kruskal-Wallis* analysis of variance (ANOVA) followed by *Dunn's-Bonferroni* test. Scale bar 100 um.
